# Dynamic profiles of SARS-Cov-2 infection from five Chinese family clusters in the early stage of the COVID-19 pandemic

**DOI:** 10.1038/s41598-020-79035-1

**Published:** 2020-12-16

**Authors:** Xiang-Gen Kong, Jin Geng, Tao Zhang, Bin Wang, An-Zhao Wu, Di Xiao, Zhao-Hua Zhang, Cai-Feng Liu, Li Wang, Xue-Mei Jiang, Yu-Chen Fan

**Affiliations:** 1 Jinan Infectious Diseases Hospital, Cheeloo College of Medicine, Shandong University, Jinan, 250021 Shandong China; 2 Department of Hepatology, Qilu Hospital, Cheeloo College of Medicine, Shandong University, Wenhuaxi Road 107#, Jinan, 250012 China; 3 Department of Biostatistics, School of Public Health, Cheeloo College of Medicine, Shandong University, Jinan, 250012 China

**Keywords:** Viral infection, Epidemiology

## Abstract

Although several cases of family clusters with SARS-Cov-2 infection have been reported, there are still limited data preventing conclusions from being drawn regarding the characteristics and laboratory findings in the COVID-19 population within family clusters. In the present study, we retrospectively collected five family clusters with COVID-19 and summarized the dynamic profiles of the clinical characteristics, laboratory findings, immune markers, treatment and prognosis of this population. Furthermore, we also compared clinical and laboratory data between the SARS-Cov-2 infection with family cluster (n = 21) and those without family cluster (n = 16). We demonstrated that the duration of SARS-Cov-2 replication might be varied based on the different family clusters due to their different genetic backgrounds. The onset improved lung radiology might start at the end of the SARS-Cov-2 positive period. Furthermore, the obtained results demonstrated that similar basic characteristics and clinical findings seem to exist between the cases with SARS-Cov-2 and without family clusters. The serum level of ferritin might have a different biological function and be a new biomarker for the family cluster. Further studies with larger numbers of patients are required.

## Introduction

Coronaviruses are members of virus family and can spread from person to person; these virus have caused pandemics of the common cold, severe acute respiratory syndrome (SARS) and Middle East respiratory syndrome (MERS) for the last decade^1^. In December 2019, a group of patients in Wuhan, China, presented with pneumonia of unknown cause, and a previously undiscovered coronavirus was immediately identified using gene sequencing^2,3^. On Feb 11, the virus was named severe acute respiratory syndrome coronavirus 2 (SARS-CoV-2), and the SARS-Cov-2 infection was named coronavirus disease 2019 (COVID-19)^4^. In March 2020, the World Health Organization (WHO) declared the COVID-19 outbreak a pandemic. To date, more than 36,000,000 SARS-Cov-2 infections have been confirmed around the world, and 1,050,374 patients have died. The development of a coronavirus vaccine is still ongoing, and the current prevention strategy usually includes frequent hand washing, social distancing and wearing masks.

SARS-Cov-2 infection in family is an important mode of transmission from person to person^5^. Chan and colleagues first reported a family cluster of SARS-Cov-2 infection with five patients diagnosed with pneumonia of unknown cause in Shenzhen^6^. A total of 11 patients in a family cluster were also reported to be infected with SARS-CoV-2 in Nanjing^7^. The clinical characteristics and laboratory parameters of COVID-19 patients in some family clusters from other Chinese cities have also been reported^8–12^. Ghinai I et al. reported an American family cluster of 16 subjects with confirmed or probable COVID-19 and demonstrated that there were three deaths due to SARS-CoV-2 infection transmitted at two family gatherings^13^. A family cluster in France with one asymptomatic case has also been reported by Danis et al., who highlighted the transmission potential of an asymptomatic individual^14^.

Although several cases of family clusters with SARS-Cov-2 infection have been reported, there are still limited data, preventing conclusions from being drawn regarding the characteristics and laboratory findings for family clusters. Jinan is the local capital city of Shandong Province, China, and has a population of more than 9 million. During the SARS-Cov-2 epidemic, in the period from January 2020 to the end of March 2020, the Jinan Infectious Diseases Hospital was the only designated tertiary hospital for COVID-19. In the present study, we retrospectively collected five family clusters with COVID-19 and summarized the dynamic profiles of the clinical characteristics, laboratory findings, immune markers, treatment and prognosis of this population. Furthermore, we also compared the clinical and laboratory data between family clusters with SARS-Cov-2 infection and without.

## Results

### General characteristics of all the included patients

There were a total of 37 confirmed patients with SARS-Cov-2 infection recorded to be in isolation in the inpatient ward of Jinan Infectious Diseases Hospital during January 28 to April 10, 2020. As shown in Table 1, the average age of all the patients was 33.76 years old with the standard derivation (SD) of 18.51 years, and the average number of days of illness before admission was 6.49 ± 4.81 days. Of all the COVID-19 patients, there were 8 (21.6%) asymptomatic individuals and 29 (78.4%) symptomatic individuals, with the most frequent symptoms being cough (n = 17, 46.0%), expectoration(n = 10, 27.0%), fever (n = 9, 24.3%), and fatigue (n = 7, 18.9%), followed by chest pain (n = 6, 16.2%), pharyngeal pain (n = 5, 14.5%), diarrhoea (n = 2, 5.4%), headache (n = 1, 2.7%) and joint pain (n = 1, 2.7%). Furthermore, a total of 20 patients (54.1%) reported visiting Wuhan. According to the guideline from the Chinese National Health Commission^15^, there were no severe cases of COVID-19 among all the patients. A total of five patients (13.5%) were defined as mild type, and the rest of the patients (n = 32, 86.5%) were defined as common type. Among all the COVID-19 patients (n = 37), a total of 21 patient (56.8%) from 5 different families were considered to be family clusters. Notably, there were no significant differences between age and sex in patients who were members or not of a family cluster (both *P* > 0.05, respectively).

**Table 1:** The basic characteristics for all the patients and the subgroup divided by family cluster.

Characteristics	All patients (N = 37)	Familial cluster		
		Yes (N = 21)	No (N = 16)	P value
Age (years) (Mean ± SD)	33.76 ± 18.51	34.0 ± 21.49	33.44 ± 14.37	0.929
Days of illness (days) (Mean ± SD)	6.49 ± 4.81	7.19 ± 4.77	5.56 ± 4.84	0.314
**Sex**				0.272
Female	20 (54.05%)	13 (61.90%)	7 (43.75%)	
Male	17 (45.95%)	8 (38.10%)	9 (56.25%)	
**Type, severity**				0.416
Mild	5 (13.51%)	2 (9.52%)	3 (18.75%)	
Common	32 (86.49%)	19 (90.48%)	13 (81.25%)	
**Symptom**				
Fever				0.49
No	28 (75.68%)	15 (71.43%)	13 (81.25%)	
Yes	9 (24.32%)	6 (28.57%)	3 (18.75%)	
Cough				0.368
No	20 (54.05%)	10 (47.62%)	10 (62.50%)	
Yes	17 (45.95%)	11 (52.38%)	6 (37.50%)	
Expectoration				0.082
No	27 (72.97%)	13 (61.90%)	14 (87.50%)	
Yes	10 (27.03%)	8 (38.10%)	2 (12.50%)	
Fatigue				0.086
No	30 (81.08%)	15 (71.43%)	15 (93.75%)	
Yes	7 (18.92%)	6 (28.57%)	1 (6.25%)	
Diarrhea				0.096
No	35 (94.59%)	21 (100.00%)	14 (87.50%)	
Yes	2 (5.41%)	0 (0.00%)	2 (12.50%)	
Headache				0.376
No	36 (97.30%)	20 (95.24%)	16 (100.00%)	
Yes	1 (2.70%)	1 (4.76%)	0 (0.00%)	
Chest pain				0.715
No	31 (83.78%)	18 (85.71%)	13 (81.25%)	
Yes	6 (16.22%)	3 (14.29%)	3 (18.75%)	
Muscle pain				0.204
No	35 (94.59%)	19 (90.48%)	16 (100.00%)	
Yes	2 (5.41%)	2 (9.52%)	0 (0.00%)	
Joint pain				0.376
No	36 (97.30%)	20 (95.24%)	16 (100.00%)	
Yes	1 (2.70%)	1 (4.76%)	0 (0.00%)	
Pharyngeal pain				0.259
No	32 (86.49%)	17 (80.95%)	15 (93.75%)	
Yes	5 (13.51%)	4 (19.05%)	1 (6.25%)	
**Coexisting conditions**				0.568
No	28 (75.68%)	15 (71.43%)	13 (81.25%)	
Diabetes mellitus	3 (8.11%)	2 (9.52%)	1 (6.25%)	
Hypertension	2 (5.41%)	1 (4.76%)	1 (6.25%)	
Heart disease	2 (5.41%)	2 (9.52%)	0 (0.00%)	
Chronic obstructive pulmonary diseases	1 (2.70%)	1 (4.76%)	0 (0.00%)	
Breast cancer	1 (2.70%)	0 (0.00%)	1 (6.25%)	
**Habit**				
Smoking				0.843
No	35 (94.59%)	20 (95.24%)	15 (93.75%)	
Yes	2 (5.41%)	1 (4.76%)	1 (6.25%)	
Alcohol drinking				0.376
No	36 (97.30%)	20 (95.24%)	16 (100.00%)	
Yes	1 (2.70%)	1 (4.76%)	0 (0.00%)	
**Exposure histroy in Wuhan**				0.666
No	17 (45.95%)	9 (42.86%)	8 (50.00%)	
Yes	20 (54.05%)	12 (57.14%)	8 (50.00%)	

### Basic and dynamic information for the family clusters of COVID-19

**Figure 1:**
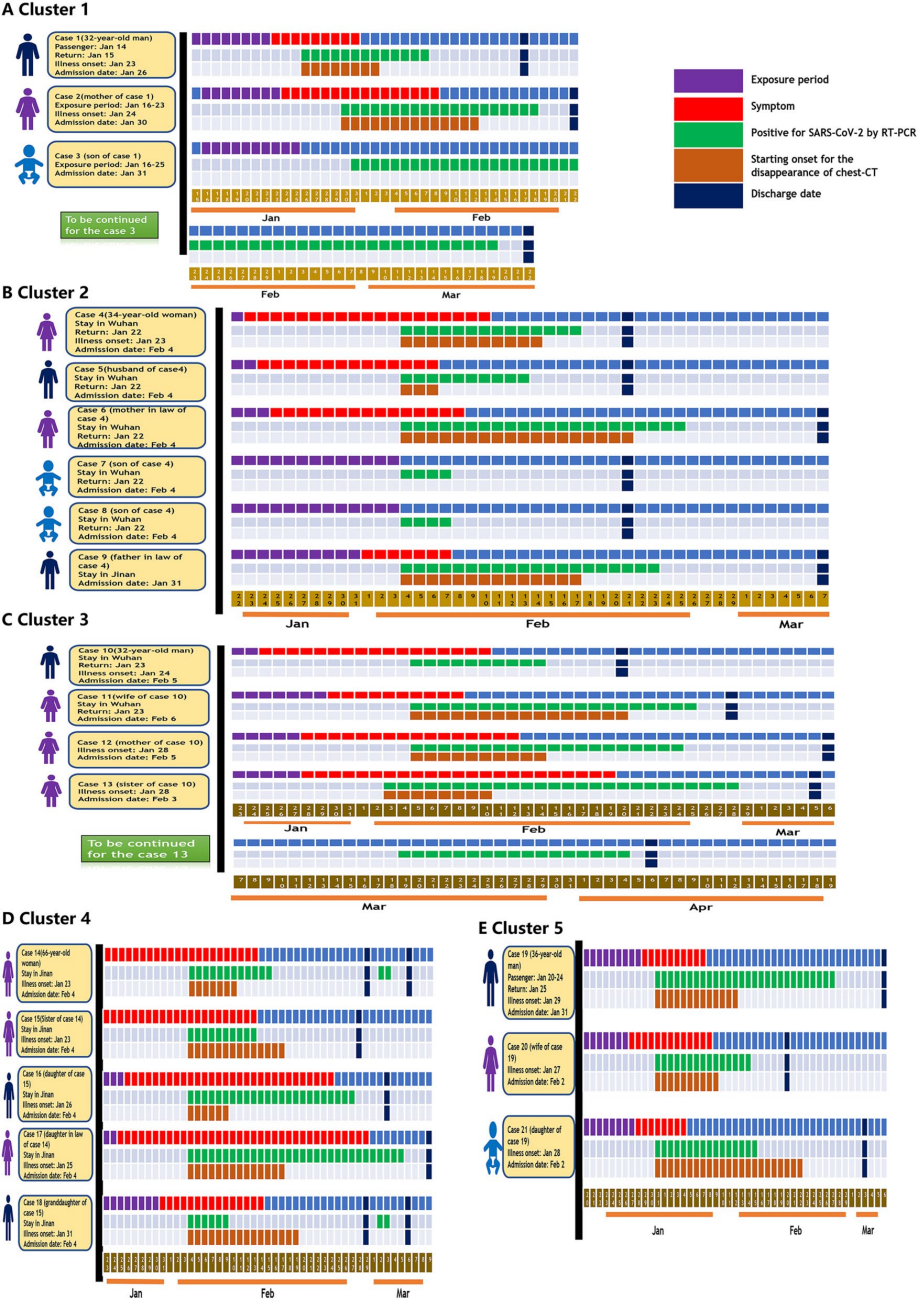
The timelines for all the 21 patients with COVID-19 from all the five family clusters.

**Figure 2:**
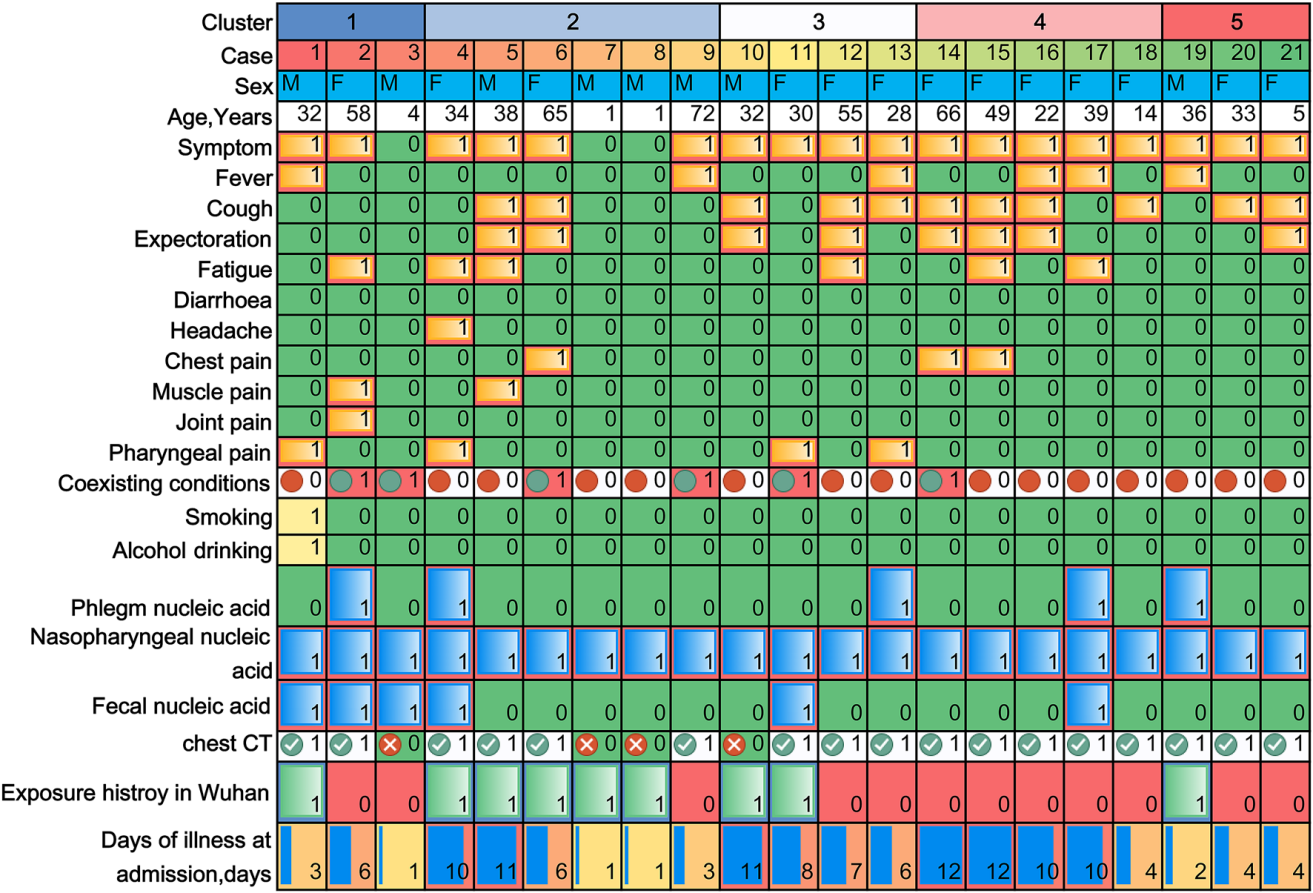
The epidemiology and basic characteristics for all the patients of the five family clusters.

There were a total of five family clusters with 21 COVID-19 patients in this study, with an average age of 34.0 ± 21.49 years old and eight male patients (38.1%). Among all 21 patients, there were three asymptomatic individuals (14.3%) and 18 symptomatic individuals (85.7%), including 11 with cough (52.4%), eight with expectoration (38.1%), six with fever (28.6%) or fatigue (28.6%), four with pharyngeal pain (19.1%), three with chest pain (14.3%), and one with joint pain or headache (4.8%). In addition, the coexisting conditions included diabetes mellitus (n = 2, 9.5%), heart disease (n = 2, 9.5%), hypertension (n = 1, 4.8%), and chronic obstructive pulmonary disease (n = 1, 4.8%). The details for each of the family clusters with COVID-19 are shown with timelines in Fig. 1 and with mixed coloured panels in Fig. 2.

### Cluster 1

On Jan 14th 2020, the index case (Case 1) was identified as a 32-year-old man who travelled to Wuhan from Chengdu, Sichuan Province. After a 1 day stay in Wuhan, he returned to Jinan (Shandong Province) on Jan 15th. Unfortunately, he began to complain of pharyngeal pain and fever, with his highest temperature recorded as for 38.0 °C on Jan 23rd 2020, and his mother (Case 2), who was a 58-year-old woman, began to feel fatigue and muscle and joint pain one day later. On Jan 26, Case 1 was admitted to our hospital and was later confirmed as SARS-Cov-2 positive, with scattered ground glass lesions in both lungs on chest CT. On Jan 30, Case 2 was confirmed to be COVID-19 by RT-PCR and chest CT. Therefore, all the close contacts were tested for SARS-Cov-2, and the son of Case 1 (Case 3, 4-year-old), who was asymptomatic, was diagnosed and hospitalized in an isolation ward on Feb 1st. The symptoms of Case 1 lasted from 9 days and disappeared on Jan 31st, and the RT-PCR results for SARS-Cov-2 were positive for 13 days and were negative twice on Feb 7th. Additionally, for Case 1, there was improvement in the lung shadow on chest CT, which started on Feb 2nd. Finally, Case 1 was discharged from the hospital on Feb 16th and had no relapse of SARS-Cov-2 after one month of follow-up. Case 2 experienced 16 days of symptoms and 20 days of SARS-Cov-2 positive status, in addition to 14 days of unchanged status of the lung shadow on chest CT. Ultimately, she was discharged from the hospital on Feb 18th. Although Case 3 had no symptoms or signs, his SASR-Cov-2 positive status lasted for approximately 49 days. He was discharged from the hospital on Mar 22nd. Fortunately, we did not find a relapse of SARS-Cov-2 infection after one month of follow-up for either Case 2 or Case 3.

### Cluster 2

This cluster included five patients with COVID-19, with four patients who had been living in Wuhan and travelled to Jinan before the onset of illness on Jan 22nd. The index case (Case 4, a 34-year-old woman) first began to report fatigue and headache with pharyngeal pain on Jan 23rd. Subsequently, the husband of Case 4 (Case 5) began to suffer from cough, expectoration, fatigue and muscle pain on Jan 24th. Meanwhile, the mother-in-law of Case 4 (Case 6) showed symptoms of cough, expectoration and chest pain beginning on Jan 25th. Both of the sons of Case 4 (11-month-old twins, Case 7 and Case 8) showed no symptoms or signs. Unfortunately, the father-in-law of Case 4, who had not travelled to Wuhan, developed fever, with the highest reported temperature of 38.0 °C on Feb 1st. On Feb 4, all five family members were detected as being positive for SARS-Cov-2 and were transferred to our isolation ward. Chest CT showed shadows in both of the lungs for Cases 4, 5 and 9, as well as a shadow plus pleural effusion for Case 6. There were no positive findings on chest CT for Cases 7 and 8, showing that they might be asymptomatic individuals. Case 4 was symptomatic for 19 days, had a SARS-Cov-2 positive status for 14 days, and had unchanged status with regard to lung shadow for 10 days. The symptoms in Case 5 disappeared 10 days after the onset of the illness, and the detection of SARS-Cov-2 was negative twice after 10 days of hospitalization, and there was improvement of lungs after 3 days of hospitalization. Interestingly, negative results of SARS-Cov-2 were demonstrated for Cases 7 and 8 on the fourth day of hospitalization. All of the above 4 patients were discharged from the hospital on Feb 21st. Case 6 presented with a symptomatic period of 15 days, SARS-Cov-2 positive testing for 22 days and a solid period of chest shadow for 18 days. Case 9 recovered after 5 days of symptoms, 20 days of testing positive for SARS-Cov-2 and 14 days of unchanged lung findings on chest CT. Both of them were discharged from the hospital on Mar 7th. There were no relapses of SARS-Cov-2 infection after one month of follow-up.

### Cluster 3

The index case (Case 10, a 32-year-old man) and his wife (Case 11, a 30-year-old woman) had been living in Wuhan and travelled to Jinan on Jan 23rd by private car. On Jan 25th, Case 10 reported cough and expectoration without fever and headache, and Case 11 reported pharyngeal pain on Jan 30. Both cases were confirmed as having SARS-Cov-2 infection and were isolated in our ward, where there was no positive finding on chest CT for Case 10 and scattered ground glass lesions for Case 11. On the same day, Jan 28th, the mother of Case 10 (Case 12, a 55-year-old woman) started to show symptoms, including cough, expectoration and fatigue. The sister of Case 10 (Case 13, a 28-year-old woman) reported fever, cough and pharyngeal pain. Case 13 was confirmed on Feb 3 and Case 12 was confirmed on Feb 5th by RT-PCR and positive findings on chest CT. During the hospitalized period, Case 10 had a symptomatic period of 17 days, was PCR positive for 10 days, and was discharged from the hospital on Feb 20th. Case 11 recovered after 10 days of symptoms, 21 days with PCR positive status, and 16 days without improvement of chest CT. On Feb 18th, Case 11 was discharged. After 16 days of symptoms and 20 days of PCR positive status, Case 12 was discharged on Mar 6th. In addition, Case 13 was discharged on Mar 5th after 23 days of symptoms, 26 days of SARS-Cov-2 positivity and 8 days without improvement on chest CT. During the follow-up, Case 13 was determined to be SARS-Cov-2 positive according to nasopharyngeal and faecal nucleic acid findings on Mar 19th. The positive status lasted for 17 days without any symptoms and signs, and Case 13 was discharged again on April 6th.

### Cluster 4

Case 14 (a 66-year-old woman) and her sister (Case 15, a 49-year-old woman) might be considered to be the index cases of this cluster since they simultaneously presented with cough, expectoration and chest pain on Jan 23rd. They had no history of travelling to Wuhan and remained in Jinan within the last 6 months. After two days, the daughter-in-law of Case 14 (Case 17) presented with a cough and fever, with the highest temperature being 38.2 °C. On Jan 26th, the daughter of Case 15 (Case 16) presented with fever, cough and expectoration. Then, the granddaughter of Case 15 (Case 18, a 14-year-old girl) developed a cough. All five family members were SARS-Cov-2 positive, with varying degrees of ground glass shadows in both lungs according to chest CT. After 22 days of symptoms, 12 days of SARS-Cov-2 positivity and 7 days of unchanged lung shadow, Case 14 was discharged from the hospital on Feb 29th. Case 15 experienced symptoms for 22 days, had SARS-Cov-2 positivity for 10 days and an unchanged lung shadow for 14 days. Case 15 was ultimately discharged on Feb 28th. For Case 16, the disease progression included a symptomatic period of 30 days, SARS-Cov-2 positivity for 24 days and a persistent size of lung shadow for 6 days. Case 16 was discharged on Mar 3rd. Case 17 presented with symptoms for 36 days, SARS-Cov-2 positivity for 31 days, and an unchanged size of lung shadow for 14 days. Case 17 was discharged on Mar 9th. Case 18 experienced symptoms for 15 days, SARS-Cov-2 positivity for 19 days and an unchanged size of lung shadow for 16 days. Case 18 was ultimately discharged on Feb 29th. After discharge, the SARS-Cov-2 results from nasopharyngeal nucleic acid testing of Case 14 and Case 18 were positive again, but for only two days. Therefore, they stayed in our isolation ward for 4 days until their SARS-Cov-2 test were negative twice and were finally discharged on Mar 6th.

### Cluster 5

The index case (Case 19, a 36-year-old man) was a local citizen of Jinan and stayed in Wuhan for 5 days from Jan 20th to Jan 25th. On Jan 25th, he came back home to Jinan to stay with his wife (Case 20, a 33-year-old woman) and daughter (Case 21, a 5-year-old girl). After two days, Case 20 reported an intermittent cough, and Case 21 presented with cough and expectoration three days later. Unfortunately, Case 19 developed fever, with the highest temperature reaching 38.9 °C on Jan 29th. All three family members were confirmed as having SARS-Cov-2 infection and were isolated in our hospital. Case 19 experienced symptoms for 10 days, 28 days of SARS-Cov-2 positive status and 13 days of no change in the size of the lung shadow. Case 19 was discharged from the hospital on Mar 6th. For Case 20, the disease progression included 13 days of symptoms, 15 days of SARS-Cov-2 positive status and 10 days of no change in the size of the lung shadow. Case 21 had a symptomatic period of 8 days, SARS-Cov-2 positive status for 16 days and no change in the size of the lung shadow for 23 days, and was ultimately discharged from the hospital on Mar 3rd.

### The characteristics of the family clusters compared with the non-family cluster

Table 1 demonstrates that age and sex were well matched between the family clusters and non-family cluster. There were no significant differences in disease severity (*P* > 0.05), coexisting conditions (*P* > 0.05), habits of smoking and alcohol drinking (both *P* > 0.05) or exposure history in Wuhan between the family clusters and non-family cluster. Furthermore, we also present the symptom distribution, including the percentage of fever, expectoration, fatigue, diarrhoea, cough, headache, chest pain, muscle pain, joint pain and pharyngeal pain in Table 1. Although there seems to be little differences in expectoration (*P* = 0.082), fatigue (*P* = 0.086) and diarrhoea(*P* = 0.096) between the family clusters and non-family cluster, the derivations were not statistically significant (all *P* > 0.05, respectively).

### The laboratory findings in the family clusters compared with the non-family cluster

Table 2 displays the laboratory findings, including routine blood tests, liver function, cardiac function and inflammatory factors, in the general population divided by the presence of family clusters. In detail, the values and the percentages of individual categories for each variable are included and compared. We did not find significant differences in routine blood tests, liver function or cardiac function (all *P* > 0.05, respectively). Regarding the inflammatory factors, the serum levels of interleukin (IL)-6, procalcitonin (PCT), C-reaction protein (CRP) and ferritin were determined. Of interest, the serum level of ferritin in COVID-19 patients without family clusters [200.95(92.53–456.07) U/L] was significantly higher (*P* = 0.043) than that of patients with family clusters [87.63(62.61–325.80) U/L]. However, we did not find a significant difference in the category of ferritin divided by 400 U/L between the two groups (*P* = 0.095).

**Table 2:** The laboratory findings for all the patients and the subgroup divided by family cluster.

Characteristics	All patient (N = 37)	Familial cluster		
		Yes (N = 21)	No (N = 16)	P value
**Blood test**				
White blood cell, E + 09/L	4.73 (3.90–5.45)	4.20 (3.69–5.05)	5.37 (4.54–6.06)	0.092
< 4	11 (29.73%)	8 (38.10%)	3 (18.75%)	0.202
≥ 4, < 10	26 (70.27%)	13 (61.90%)	13 (81.25%)	
Lymphocyte, E + 09/L	1.70 (1.20–2.00)	1.40 (1.00–2.10)	1.80 (1.70–2.00)	0.748
≥ 0, < 4	34 (91.89%)	19 (90.48%)	15 (93.75%)	0.718
≥ 4, < 8	3 (8.11%)	2 (9.52%)	1 (6.25%)	
Neutrophils, E + 09/L	2.60 (2.00–3.20)	2.60 (2.00–2.90)	2.95 (2.10–3.82)	0.139
< 2	9 (24.32%)	5 (23.81%)	4 (25.00%)	0.238
≥ 2, < 7	26 (70.27%)	16 (76.19%)	10 (62.50%)	
≥ 7	2 (5.41%)	0 (0.00%)	2 (12.50%)	
Platelet, E + 09/L	188.00 (158.00–238.00)	165.00 (149.00–205.00)	228.00 (183.75–238.75)	0.194
< 100	1 (2.70%)	1 (4.76%)	0 (0.00%)	0.623
≥ 100, < 300	33 (89.19%)	18 (85.71%)	15 (93.75%)	
≥ 300	3 (8.11%)	2 (9.52%)	1 (6.25%)	
**Liver function**				
Alanine aminotransferase (U/L)	25.22 (24.01) 18.00 (14.00–27.00)	20.00 (15.00–27.00)	16.50 (13.00–36.25)	0.69
< 40	34 (91.89%)	20 (95.24%)	14 (87.50%)	0.393
≥ 40	3 (8.11%)	1 (4.76%)	2 (12.50%)	
Aspartate aminotransferase (U/L)	25.86 (14.34) 23.00 (19.00–28.00)	23.00 (19.00–27.00)	22.50 (18.75–28.75)	0.527
< 40	34 (91.89%)	19 (90.48%)	15 (93.75%)	0.718
≥ 40	3 (8.11%)	2 (9.52%)	1 (6.25%)	
**Cardiac function**				
Lactate dehydrogenase (U/L)	212.00 (180.00–275.00)	227.00 (189.00–275.00)	202.00 (154.00–256.75)	0.458
< 245	25 (67.57%)	13 (61.90%)	12 (75.00%)	0.399
≥ 245	12 (32.43%)	8 (38.10%)	4 (25.00%)	
Creatine kinase (U/L)	97.98 (71.12) 84.10 (51.40–106.90)	88.00 (72.10–153.70)	57.40 (45.90–94.88)	0.657
< 140	29 (78.38%)	15 (71.43%)	14 (87.50%)	0.239
≥ 140	8 (21.62%)	6 (28.57%)	2 (12.50%)	
Creatine kinase isoenzymes (U/L)	14.00 (12.00–22.00)	16.00 (13.00–24.00)	13.50 (11.75–17.00)	0.494
< 25	31 (83.78%)	16 (76.19%)	15 (93.75%)	0.151
≥ 25	6 (16.22%)	5 (23.81%)	1 (6.25%)	
Myoglobin (U/L)	15.00 (10.00–25.00)	17.00 (13.00–29.00)	12.50 (8.75–16.25)	0.08
< 46	33 (89.19%)	18 (85.71%)	15 (93.75%)	0.435
46	4 (10.81%)	3 (14.29%)	1 (6.25%)	
Type B natriuretic peptide (pg/mL)	45.00 (31.00–103.00)	65.00 (45.00–116.00)	31.00 (19.00–50.75)	0.206
< 125	29 (78.38%)	16 (76.19%)	13 (81.25%)	0.711
≥ 125	8 (21.62%)	5 (23.81%)	3 (18.75%)	
**Inflammatory factors**				
Erythrocyte sedimentation rate (mm/h)	15.78 (18.06) 10.00 (3.00–20.00)	14.90 (19.44) 7.00 (4.00–20.00)	16.94 (16.63) 14.50 (2.00–21.25)	0.74
< 20	27 (72.97%)	15 (71.43%)	12 (75.00%)	0.809
≥ 20	10 (27.03%)	6 (28.57%)	4 (25.00%)	
Interlekin 6 (pg/mL)	12.36 (10.23) 9.01 (3.11–19.84)	13.39 (10.67) 10.56 (3.45–19.93)	11.02 (9.78) 7.34 (2.66–19.13)	0.492
< 7	16 (43.24%)	8 (38.10%)	8 (50.00%)	0.469
≥ 7	21 (56.76%)	13 (61.90%)	8 (50.00%)	
Procalcitonin (ng/mL)	0.05 (0.03) 0.04 (0.03–0.05)	0.04 (0.02) 0.04 (0.03–0.05)	0.05 (0.04) 0.04 (0.02–0.07)	0.135
< 0.05	26 (70.27%)	16 (76.19%)	10 (62.50%)	0.367
≥ 0.05	11 (29.73%)	5 (23.81%)	6 (37.50%)	
C-reactive protein (mg/L)	12.51 (21.53) 3.31 (0.56–12.00)	3.20 (0.27–10.20)	3.67 (0.63–14.05)	0.214
< 8.2	24 (64.86%)	15 (71.43%)	9 (56.25%)	0.338
≥ 8.2	13 (35.14%)	6 (28.57%)	7 (43.75%)	
Ferritin (U/L)	122.50 (74.20–345.40)	87.63 (62.61–325.80)	200.95 (92.53–456.07)	0.043
< 400	30 (81.08%)	19 (90.48%)	11 (68.75%)	0.095
≥ 400	7 (18.92%)	2 (9.52%)	5 (31.25%)	

### The prognosis and treatment of the family clusters compared with the non-family cluster

All of the 37 COVID-19 patients recovered and were discharged from the hospital before April 10th. The average length of admission of all the patients was 27.0 (24.0–34.0) days, with 27.0 (23.8–33.2) days for family clusters and 29.0 (25.0–29.5) days for the non-family cluster (*P* > 0.05). Table 3 demonstrates that the treatments for all the patients were follows: lipinavir/ritonavir + interferon alpha inhalation (14/37, 38.9%), lipinavir/ritonavir + interferon alpha inhalation + Peginterferon (6/37, 16.7%), and lipinavir/ritonavir + interferon alpha inhalation + antibolics (6/37, 16.7%). Furthermore, we did not find any differences in the treatments between the family clusters and non-family cluster (*P* > 0.05).

**Table 3:** The treatment regimens and prognosis for all the patients and the subgroup divided by family cluster.

Treatment and outcomes	All patients (N = 37)	Familial cluster		
		Yes (N = 21)	No (N = 16)	P value
**Antiviral treatment**				0.763
Lipinavir/ritonavir + interferon alpha inhalation	14 (38.9%)	7 (35.0%)	7 (43.8%)	
Lipinavir/ritonavir + interferon alpha inhalation + peginterferon	6 (16.7%)	3 (15.0%)	3 (18.8%)	
lipinavir/ritonavir + interferon alpha inhalation + arbidol	1 (2.8%)	1 (5.0%)	0 (0.0%)	
Arbidol + interferon alpha inhalation + ribavirin	1 (2.8%)	1 (5.0%)	0 (0.0%)	
Interferon alpha inhalation	3 (8.3%)	1 (5.0%)	2 (12.5%)	
Lipinavir/ritonavir + interferon alpha inhalation + antibolics	6 (16.7%)	3 (15.0%)	3 (18.8%)	
Interferon alpha inhalation + antibolics	2 (5.6%)	1 (5.0%)	1 (6.2%)	
Interferon alpha inhalation + ribavirin	2 (5.6%)	2 (10.0%)	0 (0.0%)	
Interferon alpha inhalation + arbidol	1 (2.8%)	1 (5.0%)	0 (0.0%)	
**Prognosis**				0.333
Admission days	27.0 (24.0–34.0)	27.0 (23.8–33.2)	29.0 (25.0–39.5)	

### The dynamic profiles of immune markers for the family clusters compared with the non-family cluster

**Figure 3:**
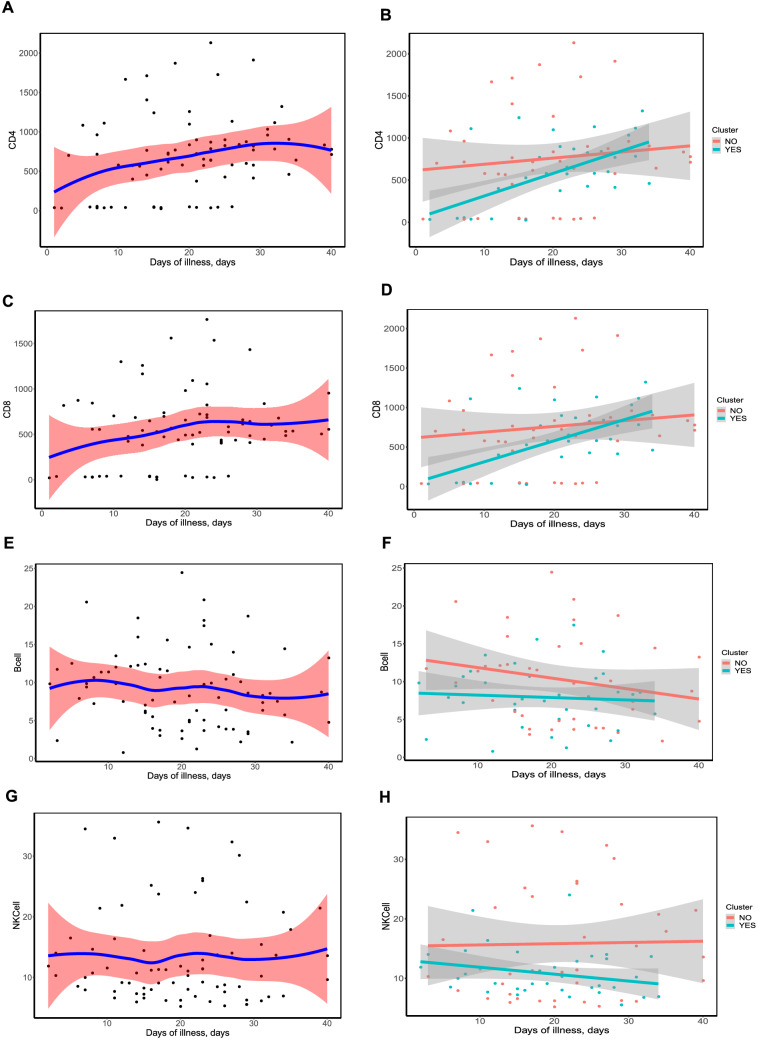
The smooth fit curves with 95% confident interval for the dynamic profiles of CD4, CD8, B cells and natural killer(NK) cells in all the patients (**A**, **C**, **E**, **G**) and the subgroup divided by family cluster (**B**, **D**, **F**, **H**).

The dynamic profiles of the percentages of immune cells has are showed as smooth curves in Fig. 3. Here, we demonstrated the increase trend of the percentages of CD4 and CD8 immune cells as the length of hospital stay increase. On the contrary, the percentage of B cells seems to show a decreasing trend according to the days of illness. In detail, we did not find any significant discrepancy among T cells, B cells and natural killing (NK) cells between the family clusters and non-family cluster.

## Discussion

The transmission of family clusters with COVID-19 accounts for the majority of SARS-Cov-2 infections worldwide^10^. However, limited data have been reported to explore the dynamic progression of COVID-19 within family clusters. Here, we summarized the characteristics of the epidemiological characteristics and disease progression style from a series of five family clusters with COVID-19 in Jinan, China. Notably, we also tried to explore the potential difference of the clinical features and possible mechanisms of immune response in the family clusters compared with the non-family cluster. As reported for the five family clusters, we demonstrated that the duration of SARS-Cov-2 replication might be varied based on different family clusters due to different genetic background. The onset of improvement of lung radiology might start at the end of the SARS-Cov-2 positive period. Furthermore, the obtained results demonstrated that similar basic characteristics and clinical findings seem to exist between the family clusters and non-family cluster. The serum level of ferritin might have a different biological function and be a new biomarker for the family cluster.

As a typical area of imported SARS-Cov-2 infection, Jinan is the local capital city of Shandong Province and has a resident population of more than 9 million people^16^. In the present study, a total of 21 COVID-19 patients from a series of five family clusters were collected, and the detailed information of disease progression were determined. The major features of these family clusters could be speculated on, and conclusion were drawn. First, without doubt, SARS-Cov-2 can be transmitted person to person. There is no specific population to be automatically immunized against the infection of SARS-Cov-2^17,18^. Our data showed that SARS-Cov-2 can infect one person regardless of the age and/or sex. Therefore, artificial isolation is a strong measure to protect from the transmission of SARS-Cov-2. Second, most of the COVID-19 cases might experience mild or common symptoms and signs and can completely recover from the SARS-Cov-2 infection^19–21^. Although there were three patients in the family clusters that showed relapse of SARS-Cov-2 replication, the duration of being positive SASR-Cov-2 usually lasts for no longer than ten days. Third, the duration of SARS-Cov-2 replication might be varied based on different family clusters due to different genetic backgrounds. In family cluster 5, all five family members showed a rather long duration of SARS-Cov-2 replication approximately 15 days. Meanwhile, in family cluster 3, two patients experienced only 4 days of a SARS-Cov-2 positive period. This discrepancy might be attributed to the varying strength of the immune response to SARS-Cov-2 on the basis of different genetic or epigenetic backgrounds^22–24^. HLA haplotypes have been reported to be associated with the disease susceptibility^25^. A recent meta-analysis revealed that 21 genes involved in toll like receptors and C-lectin pathways were associated with severe COVID-19^26^. Finally, we demonstrated that the onset of improvement of lung radiology might start at the end of the SARS-Cov-2 positive period in nearly all the family clusters. Although any increasing number of studies have revealed the mechanism of SARS-Cov-2 lung injury^27–29^, we still know little about the trend of disease progression under the same SARS-Cov-2 infection. Therefore, the interplay of virus and chest radiology should also be further studied.

The initial symptoms and signs of COVID-19 usually occur with the non-specific characteristics of the common cold or pneumonia. In the present study, there were 21.6% asymptomatic individuals and 78.4% symptomatic individuals, and the major symptoms were cough (46.0%), expectoration (27.0%), fever (24.3%) and fatigue (18.9%). Our data are in agreement with previous reports in China^21,30^. A current report from outside of China showed that impairment of taste and smell, extreme fatigue, cough, and loss of appetite were the best indicators of SARS-CoV-2 infection^31^. Impairment of taste and smell have also been reported in a small proportion of Chinese populations with SASR-Cov-2 infection^2,22,32,33^. Unfortunately, the medical records of the symptoms related to taste and smell from the COVID-19 patients in our ward were not collected.

We also demonstrated the dynamic profiles of the basic characteristics, clinical findings, treatment outcomes and immune response of COVID-19 using the family clusters compared with the age- and sex-matched non-family cluster. We did not find any differences in the basic characteristics and laboratory findings between the two groups without the severe type of COVID-19. Of interest, we reported an increased level of ferritin in COVID-19 patients without family clusters compared with those with family clusters. However, we did not find a significant difference in the category of ferritin divided by 400 U/L between the two groups. Ferritin is a blood protein that contains iron, and a high level of ferritin might indicate that excessive storage of iron in the blood. Ferritin is not a specific marker for liver disease, rheumatoid arthritis, inflammatory conditions, cancers or hyperthyroidism^2,34^. Therefore, the significance of the P value might come from the relatively small number of samples or selection bias. However, it is still helpful to further determine the po0tential role of ferritin as a functional factor or biomarker of disease progression in COVID-19 patients.

The treatment regimens and the outcome of the antiviral therapy seemed to exert similar results in the family clusters and non-family cluster, indicating that family clusters might not be a strong risk factor for the prognosis of COVID-19 patients. The assessments of peripheral immune cells might partly explain this issue. In the present study, we demonstrated the increasing trend of the percentages of CD4 and CD8 immune cells, as well as the decreasing trend accompanied by the recovery of the COVID-19 patients in the family cluster and non-family cluster. Immune function is a strong barrier against invasive pathogens. SARS-Cov-2 targets surface cells throughout the respiratory system including in the lungs; has an average incubation of six days; and has a slow disease progression^35^. The adaptive immune response may kick in before the target cells are depleted, slowing the infection and interfering with the innate immune response's ability to kill off most of the virus quickly^36–38^. Zhang et al. reported that patients with COVID-19 showed a strong interferon-α response and an overall acute inflammatory response^39^. They demonstrated that severe patients showed an altered interferon response, profound immune exhaustion with a skewed T cell receptor repertoire and broad T cell expansion^39,40^. However, the immune mechanism during the whole progression of this disease is still need.

There are some limitations of the present study. First, there is a relatively small number of samples, with a total of 21 patients from five family clusters. To date, there is not a large amount of family clusters available for exploring the specific progression style in such populations. Second, we summarized the details of the disease progression in COVID-19 patients with family clusters, as well as performed a comparative analysis with the non-family cluster to find possible discrepancies. We did not analyze genetic or epigenetic material using sequencing or molecular experiments. The molecular mechanism for the genetic or epigenetic material for the SARS-Cov-2 infection should be studied in the future.

In conclusion, we demonstrated that the duration of SARS-Cov-2 replication might be varied based in different family clusters due to their different genetic backgrounds. The onset of improvement onset in lung radiology might start at the end of the SARS-Cov-2 positive period. Furthermore, the obtained results demonstrated that similar basic characteristics and clinical findings seem to exist between the family clusters and non-family cluster. The serum level of ferritin might have a different biological function and be a new biomarker for the family clusters. Further study with a larger number of patients is needed.

## Methods

### Data sources

This retrospective study was performed in patients of COVID-19 using real-time polymerase chain reaction (RT-PCR) or gene sequencing from one isolation inpatient ward of Jinan Infectious Hospital from January 28, 2020, to April 10, 2020. The criteria for the diagnosis and treatment were based on the guidelines from the Chinese National Health Commission^15^. Epidemiological data, including a travel history to Wuhan within 14 days before the onset of illness, and a history of close contact with patients who were confirmed or suspected as having SARS-Cov-2 infection, were gathered. The exposure period was defined as the number of days from exposure to the onset of the presence of symptoms or signs for symptomatic individuals, or to the onset of SARS-Cov-2 positivity for asymptomatic individuals. Furthermore, a family cluster was defined as no less than three infections of SARS-Cov-2, and the index patient was speculated as the confirmed COVID-19 patient who had a history of travelling to Wuhan and/or who had the first symptoms and signs of pneumonia of unknown cause in the family. Data including basic characteristics, clinical symptoms and signs, laboratory results, chest radiological information, frequency for immune cell subgroups and details of treatment and prognosis, were extracted from medical records. This study conformed to the ethical guidelines of the Declaration of Helsinki and was approved by the Ethics Commission of Jinan Infectious Diseases Hospital, Cheeloo College of Medicine, Shandong University with a waiver of informed consent (2020-JC-07).

### Laboratory findings for patients with SARS-Cov-2 Infection

Specimens including sputum, stool, and nasopharyngeal swabs were obtained and the presence of SARS-Cov-2, were confirmed using a RT-PCR assay (Shanghai BioGerm Medical Biotechnology Co., Ltd, Shanghai, China) according to the published protocol^5^. The SARS-Cov-2 specific antibodies from serum were determined using the New Coronavirus Antibody Detection Kit” (Innovita Biological Technology Co., Ltd, Beijing, China) in accordance with the guideline^5^. The serum biochemical markers (COBAS integra 800, Roche Diagnostics, Germany) including aspartate aminotransferase, alanine aminotransferase, lactate dehydrogenase, creatine kinase, creatine kinase isoenzymes, and myoglobin were collected. Haematological markers (Sysmex XE-2100, Sysmex Corporation, Kobe, Japan) included white blood cells, lymphocyte, neutrophils and platelet counts. The serum concentrations of type B natriuretic peptide, IL-6, PCT and CRP were determined using the relevant enzyme-linked immunosorbent assays according to the standard protocol. The serum ferritin concentration was detected by an immunoradiometric assay. All the methods were carried out in accordance with standard regulations in the Clinical Laboratory of Jinan Infectious Diseases Hospital, Cheeloo College of Medicine, Shandong University.

### Statistical analysis

We first determined the basic characteristics and diseases progression styles for each cluster of the five families with COVID-19 (n = 21), and then compared the clinical and laboratory data between the family cluster and non-family cluster (n = 16) in the same inpatient ward. The data are expressed as percentages (%) for dichotomous variables and as medians [interquartile range (IQR)] for continuous variables. The Mann–Whitney U-test and the χ^2^-test were used to compare the two groups. All analyses were performed using Empower(R) (EmpowerStats 2.20, www.empowerstats.com, X&Y solutions Inc., Boston, MA) and R (version 3.4.3, http://www.R-project.org). Statistical significance was defined as P < 0.05.
